# Modelling Predictors of Molecular Response to Frontline Imatinib for Patients with Chronic Myeloid Leukaemia

**DOI:** 10.1371/journal.pone.0168947

**Published:** 2017-01-03

**Authors:** Haneen Banjar, Damith Ranasinghe, Fred Brown, David Adelson, Trent Kroger, Tamara Leclercq, Deborah White, Timothy Hughes, Naeem Chaudhri

**Affiliations:** 1 School of Computer Science, University of Adelaide, Adelaide, South Australia, Australia; 2 The Department of Computer Science, King AbdulAziz University, Jeddah, Saudi Arabia; 3 Auto-ID Lab, School of Computer Science, University of Adelaide, Adelaide, South Australia, Australia; 4 School of Molecular and Biomedical Science, The University of Adelaide, Adelaide, Australia; 5 Cancer Theme, South Australian Health and Medical Research Institute (SAHMRI), Adelaide. South Australia, Australia; 6 University of Adelaide, Discipline of Medicine, Adelaide, South Australia, Australia; 7 University of Adelaide, Discipline of Paediatrics, Adelaide, South Australia, Australia; 8 Centre for Cancer Biology, University of South Australia, Adelaide, South Australia, Australia; 9 Centre for Personalised Cancer Medicine, University of Adelaide, Adelaide, South Australia, Australia; 10 Haematology Department, SA Pathology, Adelaide, South Australia, Australia; 11 King Faisal Specialist Hospital and Research Centre, Oncology Center, Riyadh, Saudi Arabia; University of Thessaly Faculty of Medicine, GREECE

## Abstract

**Background:**

Treatment of patients with chronic myeloid leukaemia (CML) has become increasingly difficult in recent years due to the variety of treatment options available and challenge deciding on the most appropriate treatment strategy for an individual patient. To facilitate the treatment strategy decision, disease assessment should involve molecular response to initial treatment for an individual patient. Patients predicted not to achieve major molecular response (MMR) at 24 months to frontline imatinib may be better treated with alternative frontline therapies, such as nilotinib or dasatinib. The aims of this study were to i) understand the clinical prediction ‘rules’ for predicting MMR at 24 months for CML patients treated with imatinib using clinical, molecular, and cell count observations (predictive factors collected at diagnosis and categorised based on available knowledge) and ii) develop a predictive model for CML treatment management. This predictive model was developed, based on CML patients undergoing imatinib therapy enrolled in the TIDEL II clinical trial with an experimentally identified achieving MMR group and non-achieving MMR group, by addressing the challenge as a machine learning problem. The recommended model was validated externally using an independent data set from King Faisal Specialist Hospital and Research Centre, Saudi Arabia.

**Principle Findings:**

The common prognostic scores yielded similar sensitivity performance in testing and validation datasets and are therefore good predictors of the positive group. The G-mean and F-score values in our models outperformed the common prognostic scores in testing and validation datasets and are therefore good predictors for both the positive and negative groups. Furthermore, a high PPV above 65% indicated that our models are appropriate for making decisions at diagnosis and pre-therapy. Study limitations include that prior knowledge may change based on varying expert opinions; hence, representing the category boundaries of each predictive factor could dramatically change performance of the models.

## Introduction

Chronic myeloid leukaemia (CML) is a malignant blood cancer that results in overproduction of myeloid cells in bone marrow, leading to significant increase in the number of immature white cells circulating in an affected person’s blood. The causative chromosomal translocation, known as the Philadelphia chromosome (*Ph*), in CML gives rise to the *BCR-ABL1* gene, which encodes for the constitutively active tyrosine kinase Bcr-Abl [1]. The need for personalised medicine in CML refers to multiple active tyrosine kinase inhibitor (TKI) therapies available for CML, multiple strategies utilised for frontline CML therapy, and heterogeneity in responses. The first generation TKI, imatinib (IM) (Glivec^®^), is a standard strategy used over the past decade [2], but some patients exhibit a poor response to this therapy. These patients may benefit from a second generation TKI, such as nilotinib (NIL) (Tasigna^®^) or dasatinib (DAS) (Sprycel^®^). Each of these TKIs are currently approved for use as frontline treatment in CML. Therefore, frontline CML therapy occurs via one of two major strategies: i) frontline IM or ii) frontline second generation TKIs, such as NIL or DAS[3]. Hematologic, cytogenetic, and molecular strategies for monitoring patient responses to therapies are used by European LeukaemiaNet [4]. To monitor molecular response, real-time quantitative polymerase chain reaction (RQ-PCR) is used to quantify the level of *BCR-ABL1* mRNA transcripts in peripheral blood of patients. According to the international scale, the two main molecular responses are major molecular response (MMR; *BCR-ABL1* transcript ≤ 0.10%) and complete molecular response (CMR; *BCR-ABL1* transcripts not detectable). Molecular monitoring is considered a standard guide to clinical management in CML [5, 6]. Prediction of the long-term molecular response to frontline IM in CML can support clinicians to select optimum treatment protocols for CML patients. Patients predicted not to achieve MMR at 24 months may be better treated with alternative frontline therapies, such as NIL or DAS.

The application of machine learning in medicine reduces the gap between clinical research and clinical practice. This type of model may be useful for clinicians in decision-making by warning of specific problems or providing treatment recommendations [7]. There is a need to adapt machine learning technology to deal with the high complexity of the medical domain. Coping with the complexity of cancer patient management, we used machine learning to drive hidden information and transfer evidence into practice. Incorporating prior domain knowledge and raw data into machine learning algorithms makes the best use of information from various data sources. Using machine learning algorithms as a ‘white box’ model results in easier and more interpretable mathematical models that lead to simple and clear decisions. The decision is generated on the basis of expert experience. Thus, it is important to incorporate prior knowledge to classify medical data and identify the relation between different predictive factors.

CML treatment predictive models emulate the decision-making ability of a human expert and provide recommendations for clinicians based on early prediction of patient molecular responses to specific treatment. Thus, predictive models predicting MMR to TKI therapy from a CML patient’s clinical, molecular, and blood count factors at diagnosis have the potential to support clinicians manage CML treatment more effectively. This study aimed to i) understand whether there exist rules for predicting MMR at 24 months for CML patients treated with IM from the clinical, molecular, and cell count observations collected at diagnosis and categorised based on the available knowledge and ii) build a predictive model to predict MMR for IM in CML patients with better prediction results than those obtained with predictive assays and previous scores. CML patients predicted not to achieve MMR at 24 months may be better treated with alternative frontline therapies, including second generation TKIs, such as NIL and DAS.

## Related work

A predictive factor is a patient characteristic used to predict treatment response [8]. Predictive factors related to MMR response include common molecular assays. Other factors depend on peripheral blood counts as well as molecular-based and clinical observations of the individual patient. In order to select the most effective TKI therapy at the time of diagnosis, various predictive factors in CML have been investigated to distinguish patients at increased risk of failure with IM, the first generation TKI [9–12]. Table 1 shows the current predictive assays and score systems, factors included in the score systems and methods used, target prediction, and published results.

**Table 1:** The current predictive assays and score systems, the factors included in score systems and the methods used; the target prediction and final results.

Previous methods				
Study	Factors	Method	Target prediction	Data and Results
White *et al*. [13]	OA (ng/200,000 cells)	Kaplan Meier Analysis	MMR by 60 months to IM	TIDEL I clinical trial (n = 56), High OA: 89%, and low OA: 55%
White *et al*. [14]	IC50^IM^ (μM)	Kaplan Meier Analysis	MMR by 12 months to IM	TIDEL I clinical trial (n = 116), Low IC50^IM^: 65%, and High IC50^IM^: 39%
Sokal Score, Sokal *et al*. [15]	Age, spleen Size (cm), blast (%), and platelets (10^9^/L)	Multivariate analysis of survival	Risk groups to chemotherapy	Six European and American sources (n = 813), Low 39%, intermediate 38%, and high 23%
Hasford Score, Hasford *et al*. [16]	Age, spleen size (cm), blasts (%), eosinophils (%), basophils (%)and platelets (10^9^/L)	Multivariate analysis of survival	Risk groups to interferon alpha alone	14 studies (n = 981), Low 40.6%, intermediate 44.7%, and high 14.6%
EUTOS Score, Hasford *et al*. [17]	Basophils (%) and spleen Size (cm)	Multivariate analysis of response	CCgR at 18 months to IM	Five national study group (n = 2060), Low 79%, and high 21%

Two common predictive assays, namely OCT-1 activity (OA)[13, 18, 19] and IC50^IM^[14], have been studied to distinguish CML patients likely to achieve a good molecular response to IM. CML patients with the b3a2 *BCR-ABL1* transcript type, compared to those with the b2a2 transcript, demonstrate greater survival rates, while CML patients with the p190 transcript type are classified as high risk [20, 21].

Three common prognostic scoring systems have been developed to identify CML patient risk groups: the Sokal [15], Hasford [16], and European Treatment and Outcome Study (EUTOS)[17] scores. The Sokal score is derived using age, spleen size, platelet count, and peripheral blood blasts; the Hasford score also uses peripheral blood eosinophil and basophil percentage; and the EUTOS score is based on percentage of basophils and spleen size. These three scores ascertain the level of risk for CML patients by running multivariable regression analysis. However, these scoring systems were developed in the era when chemotherapy was the only therapy available. The EUTOS score was developed to predict cytogenetic response to IM therapy and failed to predict MMR[22]. Although prognostic scores are currently used to personalise the care of CML patients by predicting response to therapy, they were developed either for identifying risk groups or for predicting cytogenetic response to therapy, but not for molecular response.

A recent study investigated the possible association between molecular response and a number of factors, such as Sokal score, age, sex, and IM dose [23], and found that female sex is a strong predictor. A recent review of biomarkers that determine prognosis in CML also presented a list of prognostic indicators at diagnosis, such as the three scoring systems, BCR-ABL1 transcript type, and OA[24]. However, to our knowledge, relations between predictive factors to predict molecular response have not previously been considered.

## Materials and Methods

### Dataset

Patients from the Therapeutic Intensification in De Novo Leukaemia (TIDEL II) [25] clinical trial were eligible for the study. Two sequential cohorts of 105 CML patients (total of 210) received IM 600 mg as the initial therapy. All patients were in chronic phase and monitored for time-dependent molecular response targets. For the long-term prediction, we considered achieving a *BCR-ABL1* transcript level ≤ 0.1% at 24 months using RQ-PCR. When CML patients failed to achieve molecular response targets, they were either dose-escalated to 800 mg IM or switched to NIL. Where intolerance or toxicity to IM was observed, patients were switched to NIL.

Patients enrolled in the TIDEL II trial were divided into two broad outcome groups: i) positive outcome (CML patients able to remain on IM and achieve target MMR at 24 months), and ii) negative outcome (CML patients who did not achieve MMR at 24 months on IM therapy). This study used inclusion and exclusion criteria; pregnant patients were excluded. Patients found to be intolerant to IM, who switched to NIL and then achieved MMR at 24 months, were removed from analysis as they could not be assessed as MMR failures to IM because they may have switched for non-biological reasons. Patients who achieved MMR on IM constituted the positive group and patients who did not achieve MMR on IM were considered the negative group, which included i) patients who did not achieve MMR at 24 months on IM; ii) patients, who had a suboptimal response to IM, switched to NIL and went on to achieve MMR at 24 months since MMR was not achieved by administering IM; and iii) patients who received IM followed by NIL and did not achieve MMR at 24 months.

### Predictive Factors

We investigated the relation between MMR at 24 months and common predictive factors in the medical literature as mentioned in the Related Work section. Table 2 shows the list of predictive factors including description, factor type, and median with range values. All clinical, molecular, and predictive assays and peripheral blood factors were collected at the time of diagnosis, as follows: i) clinical factors: age, gender, and spleen size measured in centimetres below the costal margin; ii) molecular factors: *BCR-ABL1* transcript level pre-therapy and *BCR-ABL1* transcript type; iii) predictive assays: OA and IC50^IM^; and iv) peripheral blood factors: white cell count (WCC), absolute neutrophil count (ANC), and eosinophil, basophil, monocyte, lymphocyte, platelet, and blast counts.

**Table 2:** Predictive factor descriptions, factor type and median with range values.

Factors	Description	Type	Median (Range)
**Age (years)**	Clinical factor recorded at the time of diagnosis	Continuous	49 (17–81)
**Gender**	Clinical factor recorded at the time of diagnosis	Categorical	
**Spleen (cm)**	Clinical factor measured by observation at diagnosis	Continuous	3.8 (0–30)
***BCR-ABL1* Transcript Type**	Genetic factor identified by quantitative PCR analysis to *BCR-ABL1*. Transcript b2a2 or b3a2 is distinguished only by the absence of 75 nucleotides. Both b2a2 and b3a2 occur in patients with linked polymorphisms within exon 13 (b2) and intron 13 of the *BCR* gene.	Categorical	
**OA (ng/200,000 cells)**	The OCT-1 protein activity as a protein function can be measured by uptake in the presence and absence of a specific OCT-1 inhibitor in mRNA.	Continuous	4.7 (0–16.32)
**IC50**^**IM**^ **(μM)**	Biological factor measured as the concentration of IM producing a 50% decrease in the level of p-Crkl.	Continuous	1 (0.2–4.5)
***BCR-ABL1* level pretherapy (at diagnosis)**	Real-time quantitative polymerase chain reaction (RQ-PCR) can measure the level of *BCR-ABL1* transcripts in the peripheral blood of the patient.	Continuous	107.14 (1.96–969)
**ANC (10**^**9**^ **/L)**	Biological factor (neutrophil, granulocytes) that can be measured from peripheral blood	Continuous	32.59 (0.5–219.2)
**Monocytes (10**^**9**^ **/L)**	Biological factor in white blood cells that can be measured from peripheral blood	Continuous	1.7 (0–13.02)
**Lymphocytes (10**^**9**^ **/L)**	Biological factor in white blood cells that can be measured from peripheral blood	Continuous	3.37 (0–13.9)
**Basophils (10**^**9**^ **/L)**	Biological factor in white blood cells that can be measured from peripheral blood	Continuous	2.16 (0–38.89)
**Eosinophils (10**^**9**^ **/L)**	Biological factor in white blood cells that can be measured from peripheral blood	Continuous	1.11 (0–17.81)
**WCC (10**^**9**^ **/L)**	Biological factor and important cells in CML that can be measured from peripheral blood.	Continuous	49.6 (1.1–353.50)
**Blasts (10**^**9**^ **/L)**	Biological factor that can be measured from peripheral blood	Continuous	1.27 (0–13.9)
**Platelets (10**^**9**^ **/L)**	Biological factor that can be measured from peripheral blood	Continuous	485 (91–1219)
**Sokal Score[15]**	Risk score developed in 1984	Continuous	1 (0.45–8.08)
**Hasford Score[16]**	Risk score developed in 1998	Continuous	801 (0–2137.6)
**EUTOS Score[17]**	Risk score developed in 2011	Continuous	46.94 (0–228.5)

### Impute Missing Data

Missing values are a common problem in clinical trials. Medical data is usually collected for specific purposes (diagnosis, monitoring or treatment) and medical research aims to achieve desired outcomes by designing clinical studies to test and validate specific medical hypotheses. Medical data may involve incomplete data or missing values which may be caused by a lack of information or discontinuation of study. There are three types of missing data: missing completely at random (MCAR), missing at random (MAR) and missing not at random (MNAR). In the first type, MCAR, the absence of predictive factors value is unrelated to the outcome or other predictive factors, while the second type, MAR, the missing predictive factor is not related to missing values but may be related to observed data. The third type, MNAR, is considered non-ignorable missingness where the predictive factor missing value depends on the factor itself [26]. We aimed to have high quality predictive performance on average for all future cases using the population-based model, in contrast to the construction of patient-specific models, which are influenced by the particular predictive factor of the patient case at hand. Our population-based model is commonly used to perform well on average in all future cases. Therefore, we considered 10% as the cut-off for including a predictive factor in the analysis, since removing patient data has the undesired effect of reducing patient datasets, thus affecting the performance of the learned predictive model [27]. Missing data were treated using imputation [28] and not by removing patients who might carry information for prediction from other molecular data available. In imputation techniques, missing values are replaced with values estimated from suitable statistical methods based on information available in the dataset. For example, the imputation of missing values for blood counts were derived from known values of blood count range, and the effect of missing data on estimate of variance was beyond the scope of this study. We used linear interpolation [29] values to impute continuous and categorical data. We used the impute command in SPSS to replace missing values with linear interpolation estimation values from the last valid value before the missing value and the first valid value after the missing value. The corresponding correlation values for the original dataset (with missing values) and completed dataset (without missing values) were calculated to indicate whether imputation affected the dataset.

### Reformatting Predictive Factors Using Domain Knowledge

We reformatted factor values of data stored as text, numerals, or mixed type using existing knowledge, such as standard boundaries of blood counts, domain knowledge of clinical expertise, and previous medical publications [13, 14, 30]. Although the selected machine learning techniques have the capacity to handle continuous predictor values, reformatting the data by categorising each factor in the TIDEL II dataset into subgroups assisted comprehensibility of the final predictive model [31]. For example, we categorised the value of IC50^IM^ equal to 0.5 _**μM**_ as low IC50^IM^ and reformatted the index number of the category. If the final model selected IC50^IM^ as a relevant predictive factor, we used these categories to distinguish the predictive group on test patients.

### Machine Learning Methods

The main goal was to produce a useful predictive model that is understandable for all users. Machine learning, such as SVM and neural networks, are difficult to interpret, while logistic regression and naive Bayes allow easy interpretation of results. However, the main issue with these algorithms are the strong assumptions of conditional independence between predictive factors. They also assess the contribution of each predictive factor to classification, but not the relations. The *k*-nearest neighbours[32] technique is another well-known machine leaning algorithm, but it is sensitive to local structure of data. Classification and regression trees (CART)[33] tend to be easily interpreted by clinicians. This method has the ability to learn relationships between predictive factors and molecular response. The importance of a clear predictive model stems from the need to trust the computation to predict response. In addition, clinicians need to understand model recommendations to explain the reasons for their decision [31].

CART is a binary recursive partitioning process capable of processing continuous and categorical data as predictors or outcomes. The CART mechanism produces the optimal tree after pruning based on a cost function to avoid overfitting in the maximal tree. The steps are provided below for the basic algorithm of a decision tree, previously having been described in [33]:

1 The top-down recursive and divide-and-conquer style is used to construct the tree.
2 The root node is located in the top-most node of the tree.
3 Each node denotes a test on a factor and each branch indicates an outcome of the test, where the leaf nodes represent classes. For selected factors, the data are recursively partitioned. Here, a splitting criterion called the Gini Impurity Measure is used to determine the best split in each node.
4 For given node t, the Gini index calculates the relative frequency of class c at node t as in (1):

$$Gini(t)=1-\sum_{c}{\left[p(c|t)\right]}^{2}$$ (1)

The following scenarios demonstrate the possible indication of using the Gini index measure. In the worst scenario, patient outcomes in training data using the examined split value of predictive factor are equally distributed between both classes at the node that maximises Gini value to indicate the least interesting information. However, in the best scenario, the minimum Gini value is the most interesting information for ascertaining when all patient outcomes in training data belong to one class using the examined split value of predictive factor [34].

5 The following three conditions are used to stop splitting:
  - For the given node, all the tested data belong to the same class.
  - No factors remain for splitting.
  - No tested data are left for splitting

The starting point for this paper was a predictive model developed from a training set of patient cases. We used CART for re-expressing the decision tree as a clearly expressed set of clinical prediction rules that in the (IF..Then form) to identify relation between predictive factors and patient outcomes. CART has the ability to learn complex and non-linear relationships between factors and the response. The decision tree structure represented the extracted production rules [35]. Classifying a patient using a decision tree is effected by following a path of predictive factors through the tree to one of the leaves (patient response). This path from the root of the tree to a leaf establishes conditions which must be satisfied by any patient classified by that leaf. Thus, each leaf of a decision tree corresponds to a prediction rule. These rules are easier and lead to simple and clearer decisions which are more interpretable by clinicians than ‘black box’ mathematical models, such as SVM. The decision is generated on the basis of expert experience. The prediction rules were in the form, IF A *is S1* AND B is *S2*… THEN Response is X, where A and B are the predictive factors, *S1* and *S2* are the subcategories that belong to A and B, and X is the class (achieving MMR or not achieving MMR).

### Predictive Factor Selection

Prior to using the machine learning algorithm feature selection, algorithms were often used to select a relevant subset of input features (in our problem, a subset of predictive factors to deliver a highly predictive model) [36, 37]. This is also very important in the context of healthcare costs where fewer input factors imply fewer diagnostic tests to obtain relevant predictive factors [38]. We also need to extract relations between the most related predictive factors and to understand whether prediction rules exist. We divided the feature selection process into two main types:

i) Knowledge-driven method for feature selection, such as existing medical knowledge regarding the predictive factor as an informative feature or clinical expert judgment on molecular factors associated with predicting MMR, known as manual feature selection [39], including

- Predictive assays: OA, IC50^IM^.
- Molecular predictive factors: OA, IC50^IM^, *BCR-ABL1* transcript level pre-therapy, *BCR-ABL1* transcript type.

ii) Data-driven methods for feature selection, known as automatic feature selection. We used the wrapper approach[40] where all subsets of features are evaluated using a given machine learning approach. The models resulting from the wrapper with each machine learning algorithm *i* were *i = 1*,*2*,…2^n^, where *n* is the number of predictive factors. CART was run on the training data repeatedly using those subsets where predictive factor selection was only in the root node.

### Evaluation Measurements

Performance of predictive models was measured by using a coincidence matrix. The problem was presented as a binary classification where the test outcome was positive (achieved MMR) or negative (did not achieve MMR). Results were divided into four conditions: (a) CML patients correctly identified as not achieving MMR (‘True negative’ (TN)); (b) patients achieving MMR wrongly identified as not achieving MMR (‘False negative’ (FN)); (c) patients correctly identified as achieving MMR (‘True positive’ (TP)); and (d) patients not achieving MMR wrongly identified as achieving MMR (‘False positive’ (FP)). We reported ‘accuracy, sensitivity, specificity, positive predictive value (PPV), and negative predictive value (NPV) using the following Eqs (2–6):

$$Accuracy=\frac{TP+TN}{TP+TN+FP+FN}$$ (2)

$$\begin{array}{l} Sensitivity=\frac{TP}{TP+FN} \end{array}$$ (3)

$$Specificityy=\frac{TN}{TN+FP}$$ (4)

$$PPV=\frac{TP}{TP+FP}$$ (5)

$$NPV=\frac{TN}{TN+FN}$$ (6)

All predictive models were trained to minimise misclassification rate between predicted MMR and actual MMR. In our predictive model, data were imbalanced in the positive and negative patient groups. Two measures, G-mean (geometric mean) and F-score (weighted harmonic mean of sensitivity and PPV), have often been used to assess performance of a predictive model trained on imbalanced data as in (7 and 8):

$$G-mean=\sqrt{sensitivity\;*\;Specificity}$$ (7)

$$F-score=\frac{2\;*\;Sensitivity\;*\;PPV}{Sensitivity+PPV}$$ (8)

It is important to measure the balance between sensitivity and specificity, which means the model correctly predicted both response groups (achieving MMR or not achieving MMR). We also reported PPV (probability that a patient achieving MMR was correctly predicted to achieve MMR) because high PPV means that few patients will be unpredicted, which is crucial when making decisions in diagnosis pre-therapy. The PPV was calculated from the study test data population, in which the prevalence was 48%.

### Nested Cross-Validation

In traditional cross-validation, 10% of data (1 fold) is used for testing and the remainder for training (9 folds), with training and testing performance repeated 10 times. Standard deviation of performance of the 10 predictive models was estimated, considering they were independent. The confidence interval was obtained from the mean and standard deviation at 95% confidence level. The summary of test performances calculated on unseen folds was considered as the final performance.

However, when data are scarce, an extra layer of cross-validation should be performed. Since the test set cannot be touched (it is saved to evaluate the final models), new cross-validation was conducted on the training set. This technique is known as nested cross-validation [41]. In this case, we separated the training set into training and validation (~75%). The remaining 25% was used as the testing set. The model was trained on the training set; features were selected on the validation set; and performance was evaluated on the test set. We treated inner cross-validation as part of the model fitting procedure. To avoid overfitting, we compared the difference between training performance and inner cross-validation of the selected model. This procedure may be a good estimator of error for finding the best predictive model and predictive factor selection [42]. Finally, we reported accuracy, sensitivity, specificity, PPV, NPV, G-mean, and F-score of the testing set.

### Model Selection

To compare resulting models we used the G-mean and F-score as criteria for model selection. The wrapper approach often needs evolutionary calculations, leading to extensive processing expense. Here, we ranked the resulting models based on G-mean and F-score measurements and selected the highest values.

**Fig 1:**
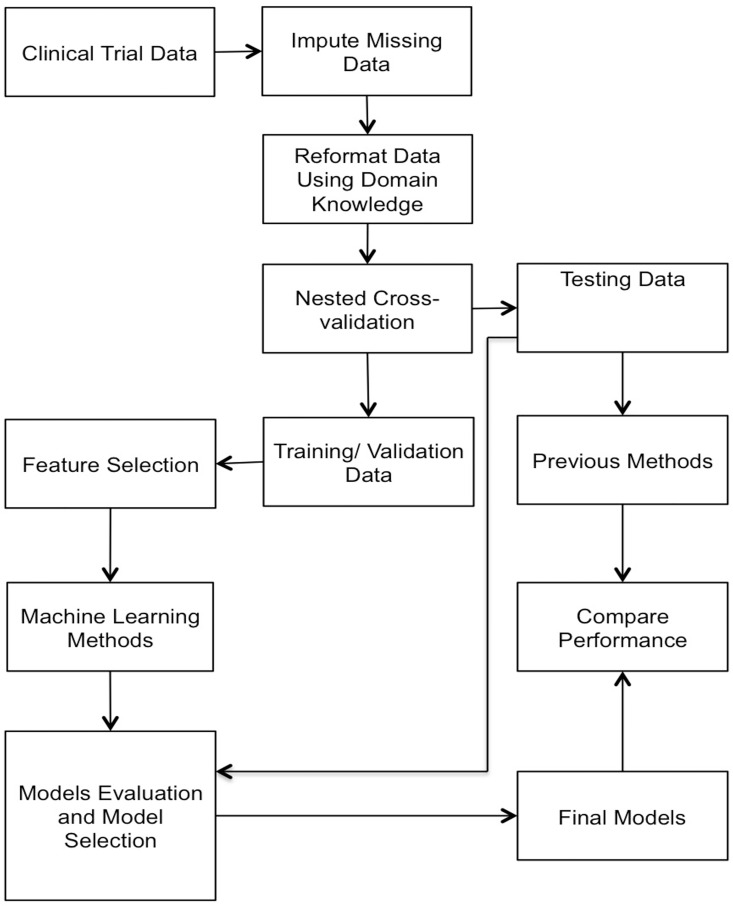
The schema for the CML predictive model, building, evaluation, and final model selection. To build the predictive model, we studied a clinical trial, preparing data for analysis by imputing missing values and reformatting factors using comprehensive standard boundaries to create subcategories for each predictive factor based on domain knowledge. For evaluation and final model selection, the nested design was used to split the dataset into training, validation and testing sets. The model was trained on the training set, features were selected on the validation set, and performance was evaluated on the test set. The final models were compared with previous methods.

The last step in our method was to compare the models generated using different feature selection techniques with current predictive assays and score systems (Table 1) based on G-mean and F-score performance to make a final recommendation. The full procedures are shown in (Fig 1), which is the schema of the CML predictive model.

### External Validation Dataset

The external dataset was obtained from a tertiary care hospital, King Faisal Specialist Hospital and Research Centre (KFSHRC), Riyadh, Saudi Arabia. There were 172 adult CML patients who used frontline TKI[43]. Only patients using frontline IM with observed MMR at 24 months were selected. We performed pre-process steps to prepare the dataset for validation. We applied inclusion and exclusion criteria, imputed missing values and reformatted predictive factors using domain knowledge. We used evaluation measurements on predicted response by the recommended model versus observed response: accuracy, sensitivity, specificity, PPV, NPV, G-mean, and F score.

### Ethics Statement

The data were analysed anonymously. All study participants provided written informed consent prior to participation. The TIDEL II trial is registered at www.ANZCTR.org.au as ACTRN12607000325404 and funded by Novartis Australia. The TIDEL II was carried out with the approval of human research ethics committees (RAH Protocol No 070718c, and ethically approved by the National Statement on Ethical Conduct in Human Research (NHMRC), 2007 and in accordance with the Declaration of Helsinki. The KFSHRC data ethically approved by Clinical Research Committee (CRC) and Research Ethics Committee (REC), 2005, Research Advisory Council (RAC) reference: ORA/1811/26, Proposal No 2051056.

## Results

In this section, we first report the number of patients for various outcomes in the full TIDEL II dataset. Then, we describe data preparation for analysis, including imputing missing values and reformatting predictive factors using domain knowledge. We reveal predictive performance of the machine learning technique and the effect of feature selection methods on selecting the final model. We also present evaluation results on unseen data based on a comparison with previous methods. Finally, we demonstrate the strong predictive factors associated with MMR at 24 months and extract the rules for prediction.

### Insight into the Data

**Fig 2:**
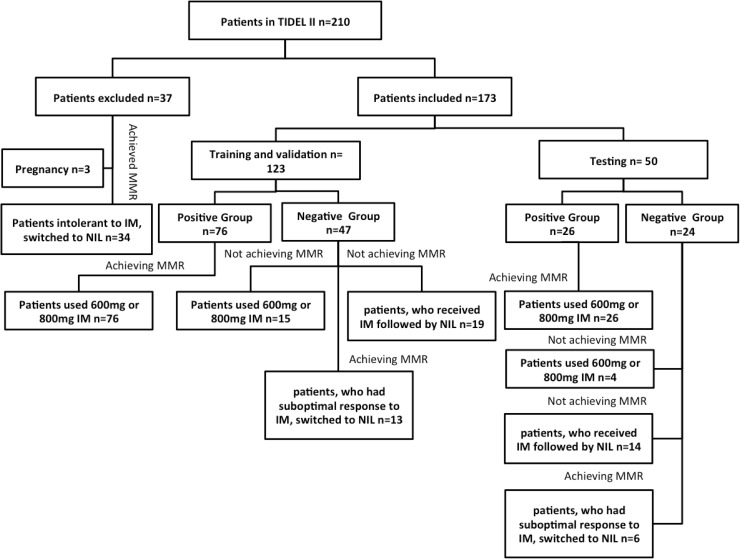
TIDEL II patients in this study. Inclusion and exclusion criteria.

In the TIDEL II clinical trial, among the 210 patients and under the inclusion/exclusion criteria, analysis included 173 (82.3%) CML patients. A positive outcome was observed in 102 (48.5%) patients able to remain on IM and achieve the target of MMR at 24 months (positive outcome), compared with 71 (33.8%) patients showing a negative outcome. The remaining 37 (34.7%) patients were excluded from analysis due to pregnancy or intolerance to IM, who were switched to NIL and then achieved MMR at 24 months. We split the TIDEL II cohort into training and testing sets. The training set included 123 (58.5%) patients and was used for inner cross-validation; the remaining 50 (23.8%) patients (testing set) were utilised for comparison between published predictive methods and our final models. The positive group was comprised of 76 training (74%) and 26 testing (26%) patients, while the negative group was compromised of 47 training (66%) and 24 testing (34%) patients. The 71 patients in negative group included i) 15 training and 4 testing of 19 (11%) patients who did not achieve MMR at 24 months on IM; ii) 13 training and 6 testing of 19 (11%) patients who had suboptimal response to IM, were switched to NIL and went on to achieve MMR at 24 months as MMR was not achieved by administering IM; and iii) 19 training and 14 testing of 33 (19%) patients who received IM followed by NIL and did not achieve MMR at 24 months. Additional details about the number of patients in the study and inclusion and exclusion criteria are shown in Fig 2.

### Imputation for Missing Values

Correlation coefficient results obtained with original data (with missing values) and complete data (missing values imputed by linear interpolation) are presented in (Tables A-C in S1 Text). We used the complete dataset for analysis. In TIDEL II, missing values were <10% as well as the external validation dataset.

### Reformat Predictive Factors Using Domain Knowledge

Knowledge was derived from standard boundaries of blood counts, clinical expertise, and previous medical publications [13, 14, 30]. The categories for each predictive factor used to transform data into categorical data and number of patients are shown in Table 3.

**Table 3:** The categories for each predictive factor used to transform data into categorical data and number of patients.

Factors	Categories	Patient in TIDEL II		Patient in Saudi Population	
		No. of patient	Patient %	No. of patient	Patient %
**Age (years)**	Young ≤30	21	12.21%	36	33.03%
	Middle Age>30, ≤60	104	60.47%	67	61.47%
	Older>60	47	27.33%	6	05.50%
**Gender**	Male	92	40.70%	45	41.28%
	Female	118	59.30%	64	58.72%
**Spleen (cm)**	Not palpable ≤1	99	57.56%	52	47.71%
	Small >1, ≤10	44	25.58%	34	31.19%
	Large >10	28	16.28%	23	21.10%
***BCR-ABL1* Transcript Type**	b2a2	68	39.53%	None	None
	b3a2	68	39.53%	None	None
	Both	34	19.77%	None	None
	e1a2	2	1.16%	None	None
**OA (ng/200,000 cells)**	Low ≤4	80	46.51%	None	None
	Standard >4	87	50.58%	None	None
**IC50**^**IM**^ **(μM)**	Group 1 ≤0.5	19	11.05%	None	None
	Group 2 >0.5 ≤0.7	31	18.02%	None	None
	Group 3 >0.7 ≤0.95	31	18.02%	None	None
	Group 4 >0.95	79	45.93%	None	None
***BCR-ABL1* level pretherapy (at diagnosis)**	Low ≤20	8	4.65%	None	None
	Moderate>20, ≤100	96	55.81%	None	None
	High>100	66	38.37%	None	None
**ANC (10**^**9**^ **/L)**	Low <1.8	3	1.74%	None	None
	Normal ≥1.8, ≤7.5	35	20.35%	None	None
	High >7.5, ≤ 50	97	56.40%	None	None
	Very High >50	36	20.93%	None	None
**Monocytes (10**^**9**^ **/L)**	Low <0.2	16	9.30%	12	11.01%
	Normal ≥0.2, ≤0.8	46	26.74%	23	21.10%
	High >0.8	109	63.37%	74	67.89%
**Lymphocytes (10**^**9**^ **/L)**	Low <1	12	6.98%	None	None
	Normal ≥1, ≤3.5	98	56.98%	None	None
	High >3.5	61	35.47%	None	None
**Basophils (10**^**9**^ **/L)**	Normal ≤0.1	28	16.28%	30	27.52%
	High >0.1, ≤1	76	44.19%	10	9.17%
	Very High >1	67	38.95%	69	63.30%
**Eosinophils (10**^**9**^ **/L)**	Normal ≤0.5	91	52.91%	28	25.69%
	High >0.5	79	45.93%	81	74.31%
**WCC (10**^**9**^ **/L)**	Low <10	29	16.86%	None	None
	Normal >10, <100	122	70.93%	None	None
	High >100	20	11.63%	None	None
**Blasts (10**^**9**^ **/L)**	Normal >0, ≤5	154	89.53%	99	90.83
	High >5	13	7.56%	10	9.17%
**Platelets (10**^**9**^ **/L)**	Low ≥20, <150	3	1.74%	8	7.34%
	Normal >150, ≤400	91	52.91%	53	48.62%
	High >400	75	43.60%	48	44.04%
**Sokal Score**	Low, intermediate≤1.2	132	79.51%	92	84.40%
	High>1.2	34	20.48%	17	15.60%
**Hasford Score**	Low, Intermediate<1480	154	93.9%	101	92.66%
	High≥1481	10	6%	8	7.34%
**EUTOS Score**	Low<87	140	83.33%	92	84.40%
	High≥87	28	16.67%	17	15.60%

Each predictive factor and the number of CML patients included in the study. Haematologist experts and previous publications identified the categories.

### Predictive Factor Selection and Prediction Results

The machine learning algorithm was trained for each feature selection method, once with all features, once with molecular features, and once with all subsets using the wrapper approach. In the wrapper approach, all subsets were trained to include different predictive factors. Here, model selection criteria were accuracy, G-mean, and F-score, which resulted from inner cross-validation performance on the training set. Predictive performance of feature selection methods with the machine learning technique on the training set is provided in Table 4. In the table, training performance and inner cross-validation performance are also presented to show fit of the models. Models A, B, and C have overfitting problems where training accuracy (A: 81%, B: 64%, C: 70%) is much larger than the cross validation mean of accuracy (A: 51%, B: 57%, C: 50%). However, the three models selected based on achieving high cross-validation mean performances (accuracy, G-mean, F-score) achieved training accuracy (D: 78%, E: 77%, F: 73%), compared with the cross-validation mean of accuracy (D: 76%, E: 75%, F: 76%); therefore, these were better models.

**Table 4:** Predictive performance of feature selection methods with the machine-learning technique from nested cross-validation methods, wrapper method used the highest cross validation performances for selecting the final models.

Feature selection approaches	Model name	Features	Training Performance							Cross-validation Performance						
			Accuracy	Sensitivity	Specificity	PPV	NPV	G-mean	F-score	Accuracy	Sensitivity	Specificity	PPV	NPV	G-mean	F-score
**All features**	**A**	1	0.81	0.83	0.77	0.86	0.72	0.80	0.84	0.51(0.43,0.59)	0.60 (0.53,0.67)	0.31(0.17,0.45)	0.63(0.49,0.77)	0.33(0.15,0.51)	0.37(0.20,0.54)	0.61(0.46,0.76)
**Molecular features**	**B**	8,9	0.64	0.67	0.54	0.80	0.38	0.60	0.72	0.57(0.5,0.64)	0.62 (0.55,0.69)	0.42(0.23,0.61)	0.75(0.63,0.87)	0.28 (0.15,0.41)	0.46(0.29,0.63)	0.67(0.55,0.79)
	**C**	8,9,10,16	0.70	0.73	0.64	0.81	0.53	0.68	0.76	0.50(0.38, 0.62)	0.60 (0.48,0.72)	0.31(0.17,0.45)	0.59(0.43,0.75)	0.36(0.16,0.56)	0.38(0.21,0.55)	0.59(0.43,0.75)
**The highest Cross-validation**																
**Accuracy**	**D**	2,3,7,13,15	0.78	0.77	0.81	0.92	0.57	0.79	0.83	**0.76(0.62,0.78)**	0.78 (0.71,0.85)	0.71(0.51,0.91)	0.88(0.78,0.98)	0.57(0.39,0.75)	0.71(0.54,0.88)	0.82(0.65,0.99)
**G-mean**	**E**	2,3,6,7,8,10,15,16	0.77	0.77	0.75	0.88	0.59	0.76	0.82	0.75(0.69,0.81)	0.79(0.72,0.86)	0.75(0.61,0.89)	0.85 (0.76,0.94)	0.59(0.42,0.76)	**0.76(0.67,0.85)**	0.81(0.69,0.93)
**F-score**	**F**	3,7,8,15	0.73	0.72	0.82	0.94	0.40	0.77	0.81	0.76 (0.68,0.84)	0.78(0.71,0.85)	0.71(0.51,0.91)	0.88(0.78,0.98)	0.57(0.39,0.75)	0.71(0.54,0.88)	**0.83(0.75,0.91)**

The features indexes are: 1 = all feature, 2 = Age, 3 = Spleen Size, 4 = Platelets, 5 = Basophils, 6 = Eosinophils, 7 = Blast, 8 = OA, 9 = IC50IM, 10 = BCR-ABL1 transcript level pre therapy, 11 = WCC, 12 = ANC, 13 = Monocytes, 14 = Lymphocytes, 15 = Gender, and 16 = BCR-ABL1 Transcript type. For each model the table gives the training and Cross-validation Performances. In cross validation performance, the means obtained from 10-fold cross validation and 95% confidence intervals.

### Comparison of Final Models and Previous Methods

We measured performance on the testing set of single predictive assays commonly used to predict MMR (OA and IC50^IM^) and common prognostic risk scores (Sokal, Hasford, EUTOS) (Table 5). OA achieved the highest accuracy (68%) compared with other previous methods (IC50^IM^: 54%, Sokal: 58%, Hasford: 56%, EUTOS: 52%). However, Model D (accuracy: 72%) outperformed OA (accuracy: 68%). Although, the Hasford score accurately predicted the positive group (those that achieved MMR at 24 months) with a sensitivity of 92%, and IC50^IM^ accurately predicted the negative group (those that did not achieve MMR at 24 months) with a specificity of 75%, G-mean (IC50^IM^: 50%, Hasford: 39%) did not exceed that of OA (67%). The three scores achieving high sensitivity (Sokal: 84%, Hasford: 92%, EUTOS: 84%) are therefore good predictors of the positive group. The highest G-mean and F-score values among previous methods were achieved by OA (G-mean: 67%, F-score: 69%). On the other hand, Models D, E, and F had G-mean and F-score values that outperformed OA performance. In addition, our models achieved PPV values better than previous methods. The PPV performances in our models (A: 73%, B: 84%, C: 80%, D: 88%, E: 84%, F: 96%) were higher than PPV in OA (67%). These high PPV values indicated that our models could be trusted in making decisions at diagnosis pre-therapy.

**Table 5:** The comparison between previous methods and, our predictive models.

		Testing Performance						
		Accuracy	Sensitivity	Specificity	PPV	NPV	G-mean	F-Score
**Previous Methods**	OA[13]	**0.68**	0.73	0.62	**0.67**	**0.68**	**0.67**	**0.69**
	IC50^IM^[14]	0.54	0.34	**0.75**	0.60	0.51	0.50	0.43
	Sokal score[15]	0.58	0.84	0.29	0.56	0.63	0.49	0.67
	Hasford Score[16]	0.56	**0.92**	0.16	0.54	0.66	0.39	0.68
	EUTOS Score[17]	0.52	0.84	0.16	0.52	0.50	0.37	0.64
**Our Model s**	Model A	0.60	0.59	0.61	**0.73**	0.45	0.60	0.65
	Model B	0.62	0.59	0.69	**0.84**	0.37	0.64	0.69
	Model C	0.58	0.56	0.61	**0.80**	0.33	0.59	0.65
	Model D	**0.72**	**0.67**	**0.81**	**0.88**	**0.54**	**0.74**	**0.76**
	Model E	0.66	0.62	0.73	**0.84**	0.45	**0.67**	**0.71**
	Model F	0.64	0.59	**0.87**	**0.96**	0.29	**0.72**	**0.73**

The bolded value indicated the comparative values between our methods and the previous methods, Model A = all predictive factors, Model B = OA and IC50^IM^, Model C = OA, IC50^IM^, *BCR-ABL1* Transcript level Pretherapy and *BCR-ABL1* Transcript Type, Model D = CART algorithm with the highest accuracy value, Model E = CART algorithm with the highest G-mean value, and Model F = CART algorithm with the highest F-score value.

### Clinical Prediction Rules and Extraction of Relations between the Predictive Factors and MMR Predictions

In this section, we demonstrate two findings: i) the selected predictive factors by CART algorithm and ii) clinical prediction rules that clearly expressed the (IF..Then) conditional relationship between the predictive factors and the MMR predictions.

Firstly, the CART uses only those factors that help separate response groups, while other factors are not considered. All predictive factors were examined at each node to assess splitter effectiveness. The decision tree generated by all features (Figure A in S1 File) had eight related factors of 15 predictive factors after pruning the maximal tree: age, spleen size, platelets, eosinophil, WCC, monocytes, IC50^IM^, and *BCR-ABL1* transcript type.

**Fig 3:**
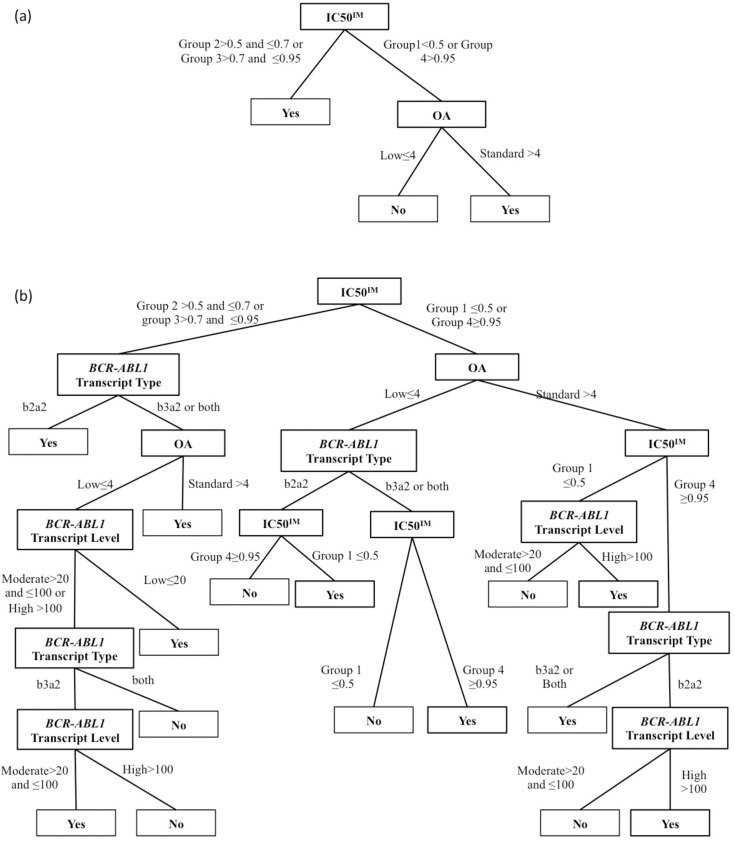
**Tree structures for a) Model B and b) Model C.** Relations between molecular predictive factors and MMR. The tree represents each predictive factor in nodes. The node has two possible splits: it is connected to either the second predictive factor or the MMR group of patients in the positive or negative group. This graphical structure illustrates the predictive rules. Predictive rules can be used on unseen data to predict the target. A predictive rule of the form: IF (conditions) THEN (class) is equivalent to a path from the root node to leaf in the decision tree, (Yes: achieve MMR at 24 months) and (No: did not achieve MMR at 24 months).

Secondly, we extracted clinical prediction rules from selected models. The rule set is attached in (S1 Table) and (Figures B and C in S1 File) represented the tree structure for model E and model F respectively. To express the conditional relationship between predictive factors and MMR predictions we presented examples from the molecular decision trees and recommended model (model D). The first example is model B and model C that used molecular predictive factors, IC50^IM^ divided patients into two groups. The groups with IC50^IM^ >0.5 **μM** and IC50^IM^ <0.95 **μM** were classified as the positive group, while for patients with IC50^IM^ <0.5 **μM** and IC50^IM^ >0.95 **μM**, OA values can help identify MMR group. In model C, adding *BCR-ABL1* transcript level and transcript type identified further relations. In the group with IC50^IM^ >0.5 **μM** and IC50^IM^ <0.95 **μM**, patients who had the b2a2 type may achieve MMR, while the other type need more information about OA and *BCR-ABL1* transcript level to identify MMR group. We also noticed that different MMR groups were identified based on the same *BCR-ABL1* transcript level. Thus, IC50^IM^, OA, and *BCR-ABL1* transcript type affected the role of *BCR-ABL1* transcript level. We also demonstrate these relations in Fig 3.

**Fig 4:**
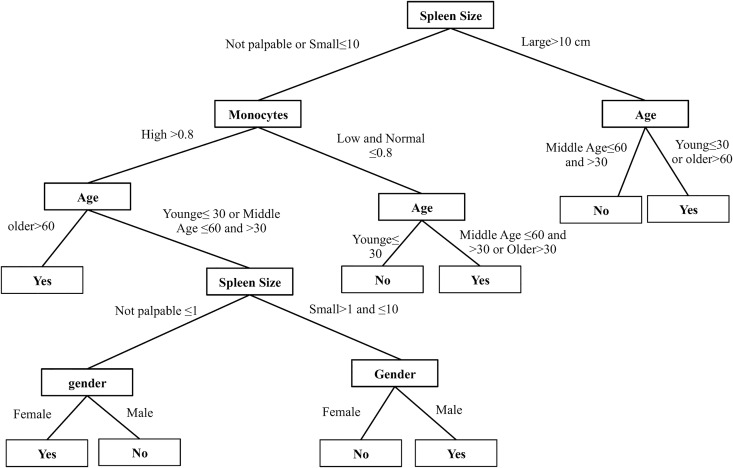
Model D structure. The final model in the tree graph that achieved high accuracy performance.

The second example is model D. Although the CART with wrapper approach recommended three models, model D showed best performance. In Fig 4, the conditional relation between age, spleen size and MMR prediction is simply structured in the tree’s right branches. For example, the first clinical prediction rule was IF spleen size belongs to the large size group >10 cm AND age belongs to the young group ≤30 or older group >60 THEN the patient may achieve MMR. On the other hand, IF spleen size belongs to the large size group >10 cm AND age belongs to the middle age group >30 and ≤60 THEN the patient may not achieve MMR. The accuracy of models measures how likely extracted rules are to correctly identify the MMR group (Table 4, training performance).

### External Validation of the Final Model

The Model D was validated in the Saudi cohort. Inclusion and exclusion criteria were applied to the dataset and the (Figure D in S1 File) shows the number of patients in the validation. Table 3 shows the number of patients included in the validation for each predictive factor. We included 109 patients who used frontline 400mg IM, while 63 (36%) patients were excluded due to missing MMR values at 24 months (27 patients) or using frontline NIL or DAS (36 patients). There were 78 (72%) patients in the positive group who achieved MMR at 24 months and 31 (28%) patients in the negative group as follows: i) 24 (77%) patients who did not achieve MMR at 24 months on 400mg IM; ii) 2 (6%) patients who had a suboptimal response to IM, switched to NIL and went on to achieve MMR at 24 months as MMR was not achieved by administering IM; and iii) 5 (17%) patients who received IM followed by NIL or DAS and did not achieve MMR at 24 months.

We compared performance of the recommended model (Model D) and common prognostic risk scores (Sokal, Hasford and EUTOS) (see Table 6). Hasford score achieved the highest accuracy (68%) compared with Sokal and our model (Sokal: 63%, Hasford: 67% and Model D: 50%). In addition, the EUTOS score achieved a slightly lesser G-mean and F-score performance than our model D (the Saudi population's G-mean: 44% vs. the EUTOS's G-mean: 41%; and the Saudi population's F-score: 29% vs. the EUTOS's F-score: 25%); furthermore, compared to the prognostic scores, our model D achieved the highest G-mean (44%) and F-score (29%) overall. Although the common prognostic risk scores achieved better accuracies, highest specificity (35%) was found in our model compared with common prognostic scores (Sokal: 13%, Hasford: 6% and EUTOS: 19%) which confirmed that model D accurately predicted the negative group (those that will not achieve MMR at 24 months) with a specificity of 35%. Validation data from the Saudi population were performed similar to testing data from TIDEL II as the three scores achieving high sensitivity (Sokal: 83%, Hasford: 92%, EUTOS: 86%) are therefore good predictors of the positive group. Although PPV value in the EUTOS score (73%) outperformed model D (68%), PPV above 60% indicated that our model could be trusted in making decisions at diagnosis pre-therapy.

**Table 6:** The comparison between previous methods and our recommended model on Saudi population.

	Validation Performance						
	Accuracy	Sensitivity	Specificity	PPV	NPV	Gmean	F-score
**Sokal score[15]**	0.63	0.83	0.13	0.71	0.24	0.33	0.17
**Hasford Score[16]**	**0.68**	0.92	0.06	0.71	0.25	0.24	0.1
**EUTOS Score[17]**	0.67	0.86	0.19	**0.73**	**0.35**	0.41	0.25
**Model D**	0.50	0.55	**0.35**	0.68	0.24	**0.44**	**0.29**

## Discussion

We employed a widely used and practical machine learning technique to develop a predictive model to support decisions related to treatment strategies for CML. Results indicate that the predictive model presented in this paper should be evaluated for potential clinical use. The early prediction of MMR at 24 months could be effective at reducing failure rate of TKI. Although the study analysed TIDEL II trial data, the methodology can be applied for different clinical trials.

Results of our study suggest that CML patients predicted not to achieve MMR at 24 months owing to IM could then be treated with alternative therapies, including the second generation TKIs NIL and DAS, or with more aggressive IM therapy, such as switching to NIL therapy and close monitoring. By contrast, CML patients predicted to achieve MMR could safely be treated with standard IM therapy with good clinical outcomes expected. The consequence of mistaken classification and subsequent treatment with IM is most likely to be treatment failure and higher risk of mortality.

OA with high G-mean accurately predicts both the positive group (a predictor for patients who will achieve MMR at 24 months) and the negative group (a predictor for patients who will not achieve MMR at 24 months). In addition, the high F-score for OA indicates it is a good predictor for the positive group (sensitivity) and can be trusted in clinical practice. However, the machine learning models often fitted the data better than previous methods (CART models fit the data better than OA). An important aspect of the decision tree is being informed by historical data and predicted unseen data so that data fits the model. However, in developing previous methods, researchers started with a model and checked whether data fit the proposed model and predictions were obtained by assuming that data are normally distributed or linearly associated. In conventional statistical methods, as data is collected by researchers to examine specific medical hypotheses or answer specific clinical questions, the approach is about a model fitting the data[44]. Thus, This predictive model was developed by addressing the challenge as a machine learning problem.

The results displayed in Tables 5 and 6 show that the small number of patients’ predictive factors included in our study may lead to overfitting and may consequently affect their generalization ability on unseen examples. We observed that the G-mean and F-score performances were reduced from 74% and 76% in the internal validation of the testing data to 44% and 29% in the external validation of the Saudi data. In the present study, it is particularly interesting to notice the difference in performance between the prognostic scores and our model D that can be beneficial for the molecular response prediction, owing to the reduced risk of misclassified patients, who will not achieve MMR at 24 months. In addition, the difference in the prediction accuracy obtained from the nested cross validation of the CART was small (Table 4), which indicates that our models were not overfit. We pruned our trees based on a cost function in order to avoid overfitting in the maximal tree. Also, we have constructed predictive models to predict MMR positive and negative groups based on the pre-therapy predictive factors (i.e. the clinical, molecular, and blood count factors) in order to learn patterns as clinical prediction rules that are associated with a response to IM.

Indeed, the recommended decision tree model D was validated internally on TIDEL II clinical trial data, and externally on the Saudi dataset. The highest G-mean, and F-score values in the testing data (TIDEL II’s G-mean: 74% and F-score: 76%) and the external validation data (Saudi population’s G-mean: 44% and F-score: 29%) compared with the prognostic scores' G-mean and F-score confirmed that our models are good predictors for positive and negative groups, while the highest sensitivities in the testing data and the external validation data were observed in prognostic scores in comparison with our model D, confirming that prognostic scores were good predictors for a positive group. As the Hasford score achieved the highest sensitivity in TIDEL II and the Saudi population (sensitivity: 92%), and the Sokal and EUTOS scores achieved higher sensitivity than our model (TIDEL II: Sokal and EUTOS scores: 84%; model D: 67%), similarly, the sensitivity of Sokal and EUTOS were higher than our model D in the external validation data (Sokal score: 83%; EUTOS score: 86%; model D score: 55%).

### Strengths and Limitations

The main strength of our study is the development of predictive models using domain knowledge in construction of clinical prediction rules which can be applied for validation on different cohorts. We extracted hidden knowledge in the form of prediction rules. Another strength is interpretability and high prediction performance of the decision tree model developed using age, gender, spleen size, blasts, and monocytes. Also, the number of predictive factors used in the model is another advantage in the context of healthcare costs as fewer input factors imply fewer diagnostic tests to obtain relevant predictive factors [38]. From the wrapper approach, predictive factors determined to be relevant predictors of MMR at 24 months were age, gender, spleen size, eosinophils, blasts, monocytes, OA, *BCR-ABL1* transcript level, and *BCR-ABL1* transcript type. Indeed, considerable evidence supports the significant impact of age[15, 16], gender[23], OA[13], spleen size[15–17], blasts, and eosinophils [15, 16].

This study is the first to investigate relations between molecular predictive factors and MMR. The levels of *BCR-ABL1* transcript pre-therapy and *BCR-ABL1* transcript type as molecular tests play a role in investigating prediction of MMR achievement at 24 months. We found that molecular factors could significantly increase model performances (Models E and F), whereas Model D could be sufficient for prediction of MMR at 24 months. This study does not ignore the significance of all predictive factors involved in the analysis because different criteria (not maximising accuracy, G-mean, and F-score) may result in different predictive factors.

We reported the performance of the three common prognostic scores in the Saudi population (KFSHRC) and TIDEL II as part of the evaluation of model performance, but this appears to be the first study to report these results, helping overcome the lack of research in the area of comparing the performance of existing prognostic scores in both Saudi and Australian populations. Although the Australia group reported their Sokal score result from TIDELL II [25], it is best to obtain the most reliable of the three prognostic performance scores and compare it with the new development model. As our results demonstrate, the previous prognostic scores are good predictors of the positive group, but our models are good predictors for both the positive and negative groups.

The tree structures were significantly influenced as the majority of patients had the same outcome. A larger dataset may increase prediction accuracy. Additionally, varying expert opinions in representing category boundaries of each predictive factor could dramatically change performance of the models.

With the available options for CML treatment, development of a method to predict CML patient response to treatment at diagnosis is critically important. We can conclude that this predictive model assists managing CML treatment by predicting the likelihood that *de novo* CML patients will achieve MMR at 24 months when treated with IM. The decision tree prediction model presented here offers medical practitioners an additional tool to provide patients with improved, individualised treatment plans. For future work, this method may also be extended to other TKIs available for use as frontline CML therapy.

## Supporting Information

S1 Text Supplementary Results. (DOCX)

S1 File Decision Tree Structures. (ZIP)

S1 Table Clinical Prediction Rules, dataset includes list of clinical prediction rules that constructed from recommended models. (XLSX)

## References

[1] Apperley JF. Part I: Mechanisms of resistance to imatinib in chronic myeloid leukaemia. Oncology. 2007;8:1018–29. 10.1016/S1470-2045(07)70342-X 17976612

[2] White DL, Hughes TP. Predicting the Response of CML Patients to Tyrosine Kinase Inhibitor Therapy. Current Hematologic Malignancy Reports 2009;4:59–65. 10.1007/s11899-009-0009-2 20425416

[3] Cortes J, Kantarjian H. How I treat newly diagnosed chronic phase CML. Blood. 2012;120:1390–7. 10.1182/blood-2012-03-378919 22613793 PMC4916560

[4] Baccarani M, Saglio G, Goldman J, Hochhaus A, Simonsson B, Appelbaum F, et al Evolving concepts in the management of chronic myeloid leukemia: recommendations from an expert panel on behalf of the European LeukemiaNet. Blood. 2006;108(6):1809–20. 10.1182/blood-2006-02-005686 16709930

[5] Hughes T, Branford S. Molecular monitoring of BCR–ABL as a guide to clinical management in chronic myeloid leukaemia. Blood. 2006;20:29–41. 10.1016/j.blre.2005.01.008 16426942

[6] Baccarani M, Deininger MW, Rosti G, Hochhaus A, Soverini S, Apperley JF, et al European LeukemiaNet recommendations for the management of chronic myeloid leukemia: 2013. 2013;122(6):872–84. 10.1182/blood-2013-05-501569 23803709 PMC4915804

[7] Cruz JA, Wishart DS. Applications of Machine Learning in Cancer Prediction and Prognosis. Cancer Informatics 2006;2:59–77. PMC2675494 19458758

[8] Oldenhuis CNAM, Oosting SF, Gietema JA, de Vries EGE. Prognostic versus predictive value of biomarkers in oncology. European Journal of Cancer. 2008;44:9 4 6–9 5 3. 10.1016/j.ejca.2008.03.006 18396036

[9] Agrawal M, Garg RJ, Kantarjian H, Cortes J. Chronic Myeloid Leukemia in the Tyrosine Kinase Inhibitor Era: What Is the “Best” Therapy? Current oncology reports (1523–3790). 2010;12(5):302–13. 20640942 10.1007/s11912-010-0116-1

[10] White DL, Hughes TP. Predicting the response of CML patients to tyrosine kinase inhibitor therapy. Current Hematologic Malignancy Reports. 2011;6(2):88–95. 10.1007/s11899-011-0087-9 21448598

[11] Guilhot F, Guilhot J. Predicting response in CML. Blood. 2011;117(6):1773–4. 10.1182/blood-2010-11-317123 21310930

[12] Jabbour E, Kantarjian H, O'Brien S, Shan J, Garcia-Manero G, Wierda W, et al Predictive factors for outcome and response in patients treated with second-generation tyrosine kinase inhibitors for chronic myeloid leukemia in chronic phase after imatinib failure. Blood. 2010;117:822–1827. 10.1182/blood-2010-07-293977 PMC4081281 21030554

[13] White DL, Saunders VA, Frede A, Dang P, Zrim S, Osborn MP, et al The Functional Activity of the OCT-1 Protein Is Predictive of Molecular Response and Survival in CP-CML Patients Treated with Imatinib: A 5 Year Update of the TIDEL Trial. Blood [0006–4971]. 2009;114(22):507–.

[14] White D, Saunders VA, Kalebic T, Hughes TP. The IC50 Assay Is Predictive of Molecular Response, and Indicative of Optimal Dose in De-Novo CML Patients. Blood [0006–4971]. 2008;112(11):405 –.

[15] Sokal J, Cox E, Baccarani M, Tura S. Prognostic discrimination in "good-risk" chronic granulocytic leukemia. Blood 1984;63:789–99. 6584184

[16] Hasford J, Pfirrmann M, Hehlmann Rd, Allan NC, Baccarani M. A New Prognostic Score for Survival of Patients With Chronic Myeloid Leukemia Treated With Interferon Alfa. Jornal of the National Cancer Institute. 1998;90(11):850–8. 10.1093/jnci/90.11.850 9625174

[17] Hasford J, Baccarani M, Hoffmann V, Guilhot J, Saussele S, Rosti G. Predicting complete cytogenetic response and subsequent progression-free survival in 2060 patients with CML on imatinib treatment: the EUTOS score. Blood. 2011;118(3):686–92. 10.1182/blood-2010-12-319038 21536864

[18] White DL, Saunders VA, Dang P, Engler J, Zannettino ACW, Cambareri AC, et al OCT-1−mediated influx is a key determinant of the intracellular uptake of imatinib but not nilotinib (AMN107): reduced OCT-1 activity is the cause of low in vitro sensitivity to imatinib. Blood. 2006;108:697–704. 10.1182/blood-2005-11-4687 16597591

[19] White DL, Saunders VA, Dang P, Engler J, Venables A, Zrim S. Most CML patients who have a suboptimal response to imatinib have low OCT-1 activity: higher doses of imatinib may overcome the negative impact of low OCT-1 activity. Blood. 2007;110:4064–72. 10.1182/blood-2007-06-093617 17761829

[20] Prejzner W. Relationship of the BCR gene breakpoint and the type of BCR/ABL transcript to clinical course, prognostic indexes and survival in patients with chronic myeloid leukemia. Medical science monitor (1234–1010). 2002;8(5). 12011769

[21] Verma D, Kantarjian HM, Jones D, Luthra R, Borthakur G, Verstovsek S, et al Chronic myeloid leukemia (CML) with P190BCR-ABL: analysis of characteristics, outcomes, and prognostic significance. Blood (0006–4971). 2009;114(11):2232–5. 19531657 10.1182/blood-2009-02-204693 PMC4828071

[22] Jabbour E, Cortes J, Nazha A. EUTOS score is not predictive for survival and outcome in patients with early chronic phase chronic myeloid leukemia treated with tyrosine kinase inhibitors: a single institution experience. Blood. 2012;119:4524–6. 10.1182/blood-2011-10-388967 22431574 PMC3362365

[23] Branford S, Yeung DT, Ross DM, Prime JA, Field CR, Altamura HK, et al Early molecular response and female sex strongly predict stable undetectable BCR-ABL1the criteria for imatinib discontinuation in patients with CML. Blood. 2013;121:3818–24. 10.1182/blood-2012-10-462291 23515925

[24] Sweet K, Zhang L, Pinilla-Ibarz J. Biomarkers for determining the prognosis in chronic myelogenous leukemia. Journal of Hematology & Oncology. 2013;6(54). 10.1186/1756-8722-6-54 PMC3737033 23870290

[25] Yeung DT, Osborn MP, White DL, Branford S, Braley J, Herschtal A, et al. TIDEL-II: first-line use of imatinib in CML with early switch to nilotinib for failure to achieve time-dependent molecular targets2015 2015-02-05 00:00:00. 915–23 p. 10.1182/blood-2014-07-590315 PMC5161008 25519749

[26] Ambler G, Omar RZ, Royston P. A comparison of imputation techniques for handling missing predictor values in a risk model with a binary outcome. Stat Methods Med Res. 2007;16(3). 10.1177/0962280206074466 17621472

[27] Acuna E, Rodriguez C. The treatment of missing values and its effect in the classifier accuracy Classification, Clustering, and Data Mining Applications. Studies in Classification, Data Analysis, and Knowledge Organisation: Springer Berlin Heidelberg; 2004 p. 639–47.

[28] Cismondia F, Fialho AS, Vieira SM, Retic SR, Sousab JMC, Finkelstein SN. Missing data in medical databases: Impute, delete or classify? Artificial Intelligence in Medicine. 2013;58(1):63–72. 10.1016/j.artmed.2013.01.003 23428358

[29] Starkweather J, Herrington R. Replace Missing Values. Secondary Replace Missing Values 2014. Available from: http://www.unt.edu/rss/class/Jon/SPSS_SC/Module6/SPSS_M6_1.htm.

[30] Thamas D. CBE What the White Cell. IMVS Newsletter. 2007.

[31] Freitas AA. Comprehensible classification models: a position paper. ACM SIGKDD Explorations. 2013;15(1):1–10.

[32] Mitchell T. Machine learning: McGraw-Hill; 1997.

[33] Breiman L, Friedman J, Olshen R, Stone C. Classification and regression trees: Belmont, Calif: Wadsworth International Group; 1984.

[34] Timofeev R. Classification and Regression Trees (CART) Theory and Applications: Humboldt University; 2004.

[35] Quinlan JR, editor Generating production rules from decision trees. Proceeding IJCAI'87 Proceedings of the 10th international joint conference on Artificial intelligence; 1987; USA: Morgan Kaufmann Publishers Inc.

[36] Perner P. Improving the accuracy of decision tree induction by feature preselection Applied Artificial Intelligence: An International Journal. 2001;15(8).

[37] Prati RC, editor Combining feature ranking algorithms through rank aggregation. Neural Networks (IJCNN), The 2012 International Joint Conference on; 2012 10–15 June 2012.

[38] Qian M, Nahum-Shani I, Murphy SA. Dynamic Treatment Regimes In: Tang W, Tu X, editors. Modern Clinical Trial Analysis. New York: Springer; 2013 p. 127–48.

[39] Cheng T-H, Wei C-P, Tseng VS, editors. Feature Selection for Medical Data Mining: Comparisons of Expert Judgment and Automatic Approaches. Proceedings of the 19th IEEE Symposium on Computer-Based Medical Systems (CBMS'06); 2006: IEEE Computer Society.

[40] Karegowda AG, Jayaram MA, Manjunath AS. Feature Subset Selection Problem using Wrapper Approach in Supervised Learning. International Journal of Computer Applications. 2010;1(7):13–7.

[41] Lasserre J, Arnold S, Vingron M, Reinke P, Hinrichs C. Predicting the outcome of renal transplantation2012 2012-03-01 00:00:00. 255–62 p. 10.1136/amiajnl-2010-000004 PMC3277611 21875867

[42] Varma S, Simon R. Bias in error estimation when using cross-validation for model selection. BMC Bioinformatics. 2006;7:91 10.1186/1471-2105-7-91 16504092 PMC1397873

[43] Khalil SH, Abu-Amero KK, Al Mohareb F, Chaudhri NA. Molecular monitoring of response to imatinib (Glivec) in chronic myeloid leukemia patients: experience at a tertiary care hospital in Saudi Arabia. Genetic testing and molecular biomarkers. 2010;14(1):67–74. Epub 2009/12/01. 10.1089/gtmb.2009.0126 19943786

[44] Shouval R, Bondi O, Mishan H, Shimoni A, Unger R, Nagler A. Application of machine learning algorithms for clinical predictive modeling: a data-mining approach in SCT. Bone marrow transplantation. 2014;49(3):332–7. 10.1038/bmt.2013.146 24096823

