# Supplementary material for: Modelling Predictors of Molecular Response to Frontline Imatinib for Patients with Chronic Myeloid Leukaemia
Source: PLoS One. 2017 Jan 3;12(1):e0168947. doi: 10.1371/journal.pone.0168947 (PMC5207707; doi:10.1371/journal.pone.0168947)
Supplement: S1 Text — (DOCX) [file pone.0168947.s001.docx]

**Modelling Predictors of Molecular Response to Frontline Imatinib for Patients with Chronic Myeloid Leukaemia**

Haneen R Banjar, Damith Ranasinghe, Fred Brown, David Adelson, Trent Kroger, Tamara Leclercq, Deborah White, Timothy Hughes, Naeem Chaudhri

**Supplement Results**

**Imputation for Missing Values**

The identifiers IDs of the patients those imputed values of the factors of patients with missing values is listed in Table A.

**Table A** The identifiers of the patients those imputed values of the factors of patients with missing values.

| **Patient ID** | **Factors with Missing value** | **Missing value imputed** |
| --- | --- | --- |
| 4 | Spleen, Sokal score, Hasford Score | 0, 0.63, 690.713 |
| 10 | IC50^IM^ | 0.6 |
| 12 | OA, IC50^IM^ | 4.02, 0.8 |
| 25 | IC50^IM^ | 0.86 |
| 26 | Eosinophils and Hasford score | 0.65, 449,91 |
| 43 | EUTOS score | 90.525 |
| 48 | Blast, Sokal Score, and Hasord Score | 0.5, 0.61, 1006.42 |
| 53 | IC50^IM^ | 0.61 |
| 64 | EUTOS score | 30 |
| 69 | IC50^IM^ | 0.85 |
| 71 | *BCR-ABL1* Level pretherapy | 48.5 |
| 77 | IC50^IM^ | 0.82 |
| 79 | IC50^IM^ | 0.87 |
| 88 | IC50^IM^ | 0.73 |
| 93 | *BCR-ABL1* Level pretherapy | 125 |
| 95 | Age, Sokal score, Hasford score | 52, 1.27, 1436.31 |
| 97 | Blast, Sokal score, Hasford score | 0, 0.76, 603.52 |
| 102 | IC50^IM^ | 2.788 |
| 124 | Platelets, Blast, Sokal score, Hasford score | 399, 0, 0.77, 661.594 |
| 126 | OA | 6.44 |
| 151 | OA, IC50^IM^ | 4.97, 0.57 |
| 153 | OA | 4.72 |
| 174 | Platelets, Blast, Sokal score, Hasford score | 311.33, 3.3, 1.22, 1250.59 |
| 175 | Platelets, Blast, Sokal score, Hasford score | 355, 1.66, 0.87, 682.16 |
| 187 | OA | 3.55 |
| 206 | IC50^IM^, EUTOS score | 1.47, 57 |
| 207 | Basophils, Eosinophils, Hasford score | 7.65, 8.905, 1024.75 |
| 208 | IC50^IM^ | 1.5 |
| 210 | EUTOS score | 57 |

The imputation is done by using the linear interpolation for the continuous and categorical data.

**Correlation Coefficients for MMR at 24 months with Original Data and Completed Data**

The original data included missing values and completed data after imputation of missing values of factors. The correlation coefficient was calculated between each predictive factor and the MMR at 24 months. Table B shows that there were not large differences in correlation coefficients.

**Table B** The correlation Coefficient in Original data and completed data

| **Original Data** | | **Completed Data** | |
| --- | --- | --- | --- |
| **Predictor** | **Correlation p** | **Predictor** | **Correlation p** |
| Spleen | 0.31521833 | Spleen | 0.31732076 |
| Age | 0.19547668 | Basophils | 0.19958623 |
| Basophils | 0.19309652 | Age | 0.19328342 |
| **Monocytes** | 0.18853493 | **WCC** | 0.18869802 |
| **WCC** | 0.17740892 | **Monocytes** | 0.18803225 |
| Eosinophils | 0.16655321 | Eosinophils | 0.18417037 |
| ANC | 0.14582059 | ANC | 0.15514001 |
| Blast | 0.110962816 | Blast | 0.12231851 |
| **IC50imatinib** | 0.10926288 | **Hasford Score** | 0.11979281 |
| **Hasford Score** | 0.108925685 | **Platelets** | 0.09735691 |
| **OA** | 0.10400609 | **IC50imatinib** | 0.096847385 |
| **Platelets** | 0.0939224 | **OA** | 0.09405362 |
| Gender | 0.08926122 | Gender | 0.08926122 |
| EUTOS Score | 0.0820541 | EUTOS Score | 0.079521775 |
| ***BCR-ABL1* level at diagnosis** | 0.03623853 | **Sokal Score** | 0.038800742 |
| **Sokal Score** | 0.03104846 | ***BCR-ABL1* level at diagnosis** | 0.030398324 |
| *BCR-ABL1* Transcript Type | 0.02592691 | *BCR-ABL1* Transcript Type | 0.02592691 |
| Lymphocytes | 0.008399159 | Lymphocytes | 0.022077713 |

**Overall Summary of Missing Values in TIDEL II and Saudi Population**

Table C Missing values percentage included in TIDEL II and Saudi datasets

| **Factors** | **TIDEL II (n= 210)** | **Saudi Population (n=172)** |
| --- | --- | --- |
| **Age (years)** | 0 | 0 |
| **Gender** | 0 | 4(2.32%) |
| **Spleen (cm)** | 1(0.48%) | 0 |
| **BCR-ABL1 Transcript Type** | 0 | Not palpable |
| **OA (ng/200,000 cells)** | 6(2.86%) | Not palpable |
| **IC50IM (μM)** | 14(6.67%) | Not palpable |
| **BCR-ABL1 level pretherapy (at diagnosis)** | 4(1.90%) | Not palpable |
| **ANC (109 /L)** | 1(0.48%) | Not palpable |
| **Monocytes (109 /L)** | 1(0.48%) | 0 |
| **Lymphocytes (109 /L)** | 1(0.48%) | Not palpable |
| **Basophils (109 /L)** | 1(0.48%) | Not palpable |
| **Eosinophils (109 /L)** | 3(1.43%) | Not palpable |
| **WCC (109 /L)** | 1(0.48%) | Not palpable |
| **Blasts (109 /L)** | 7(3.33%) | Not palpable |
| **Platelets (109 /L)** | 4(1.90%) | 9(5.23%) |
| **Blasts (% of leukocytes)** | 0 | 0 |
| **Basophils (% of leukocytes)** | 3(1.43%) | 10(5.81%) |
| **Eosinophils (% of leukocytes)** | 3(1.43%) | 10(5.81%) |

**Machine Learning Implementation**

The actual response is (*ResMMR*: Actual MMR, 1 refers to achieving MMR and 0 refers to not achieving MMR).

**Wrapper Approach**

First, the wrapper approach selects the prognostic subset (*InputFactorsIndex*: refers to the input index in the data set). For example, if the OA is located in the second column and IC50^IM^ is located in the fifth column of the data set, then the *InputFactorsIndex*=[ 2 5]. Next, the *classregtree* is a keyword in Matlab to implement (*DTStruct*) CART structure; *fitcknn* is a keyword to build KNN. In addition *NaiveBayes.fit* is reserved for building Naive Bayes. Finally, the *P* refers to the prediction results; 1: Yes, and 0: No. For construction and evaluation of the CART model:

*DTStruct* = classregtree( TrianData(*InputFactorsIndex)*, *ResMMR*, *'method'*, *'classification'*, *'splitcriterion'*, *'gdi'*, *'categorical'*,1: length(*InputFactorsIndex*));

*P*= eval(*DTStruct,TestData*(*InputFactorsIndex*));
