# Supplementary figures and images for: Modelling Predictors of Molecular Response to Frontline Imatinib for Patients with Chronic Myeloid Leukaemia
Source: PLoS One. 2017 Jan 3;12(1):e0168947. doi: 10.1371/journal.pone.0168947 (PMC5207707; doi:10.1371/journal.pone.0168947)

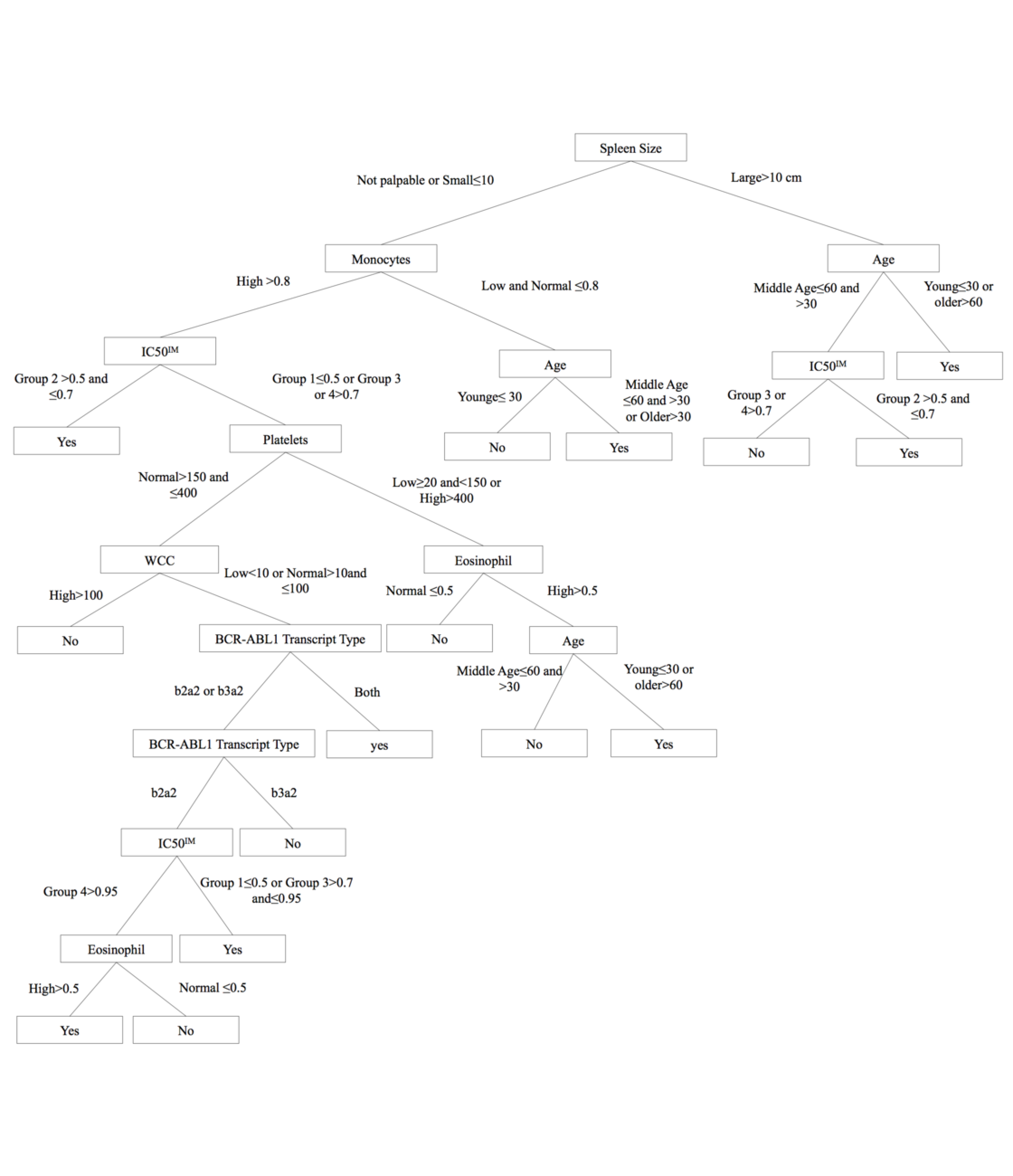

Supplement: S1 File — (ZIP) [file pone.0168947.s002.zip › S1_File/S1_FigA.tif]

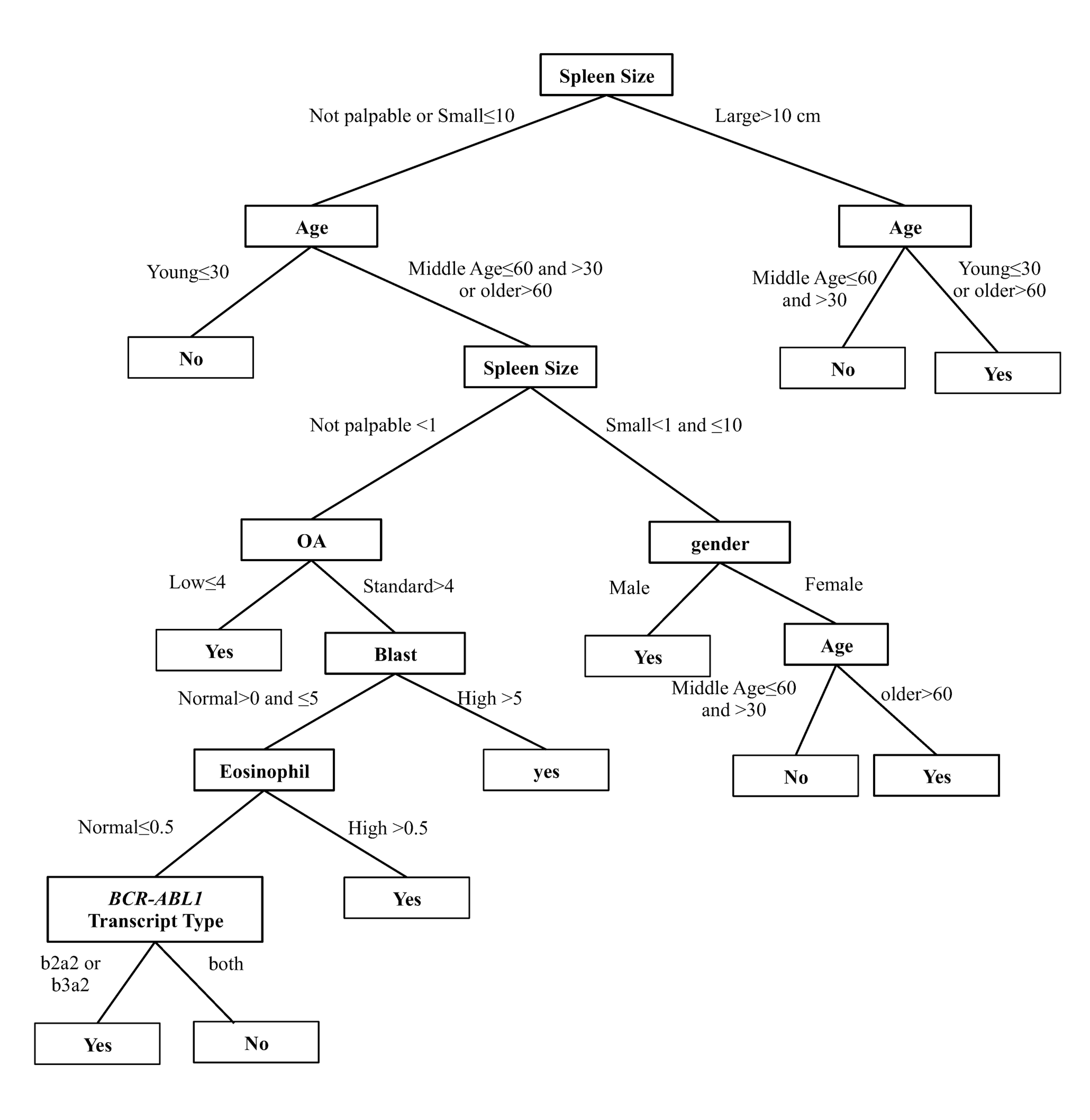

Supplement: S1 File — (ZIP) [file pone.0168947.s002.zip › S1_File/S1_FigB.tif]

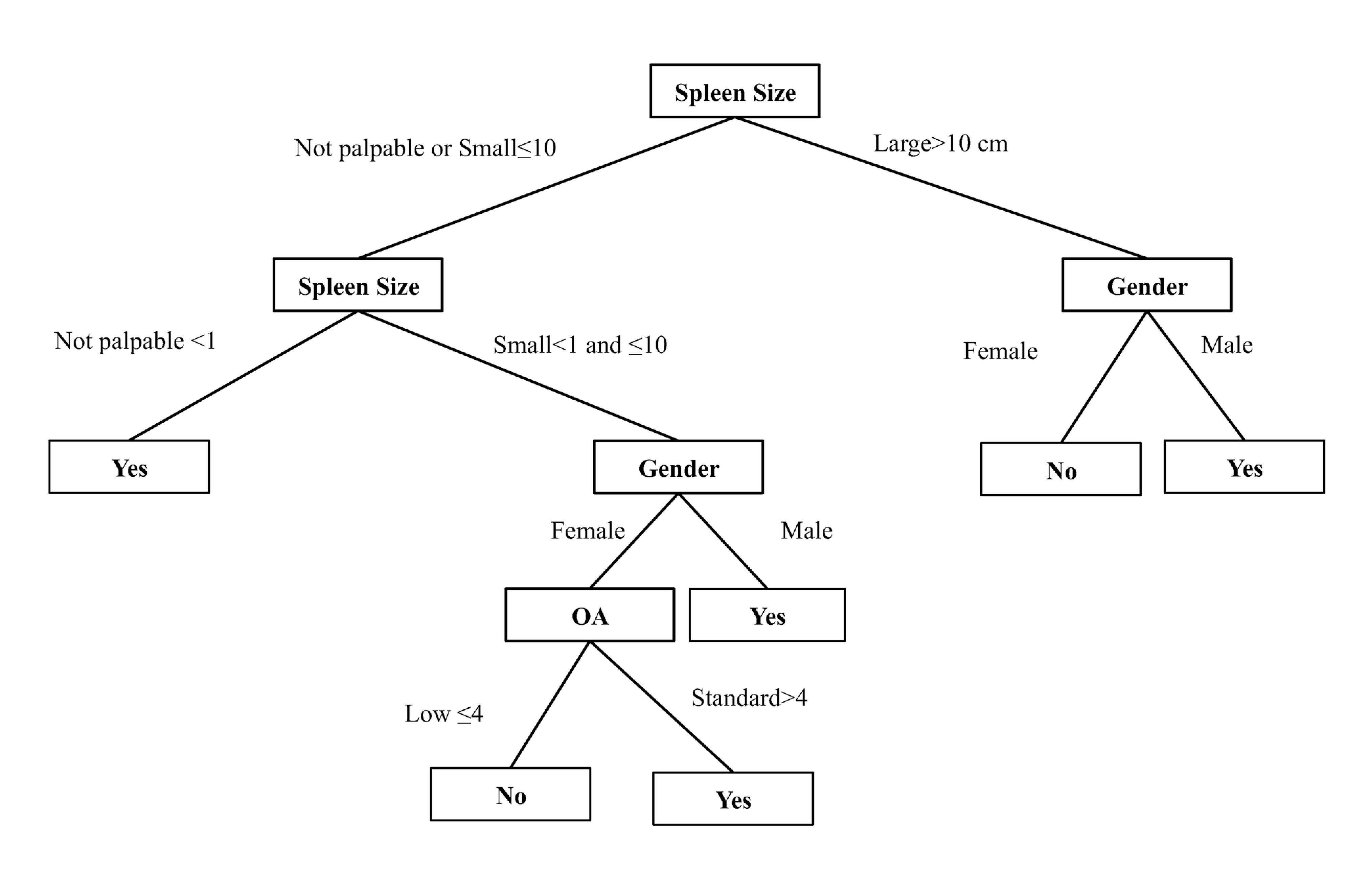

Supplement: S1 File — (ZIP) [file pone.0168947.s002.zip › S1_File/S1_FigC.tif]

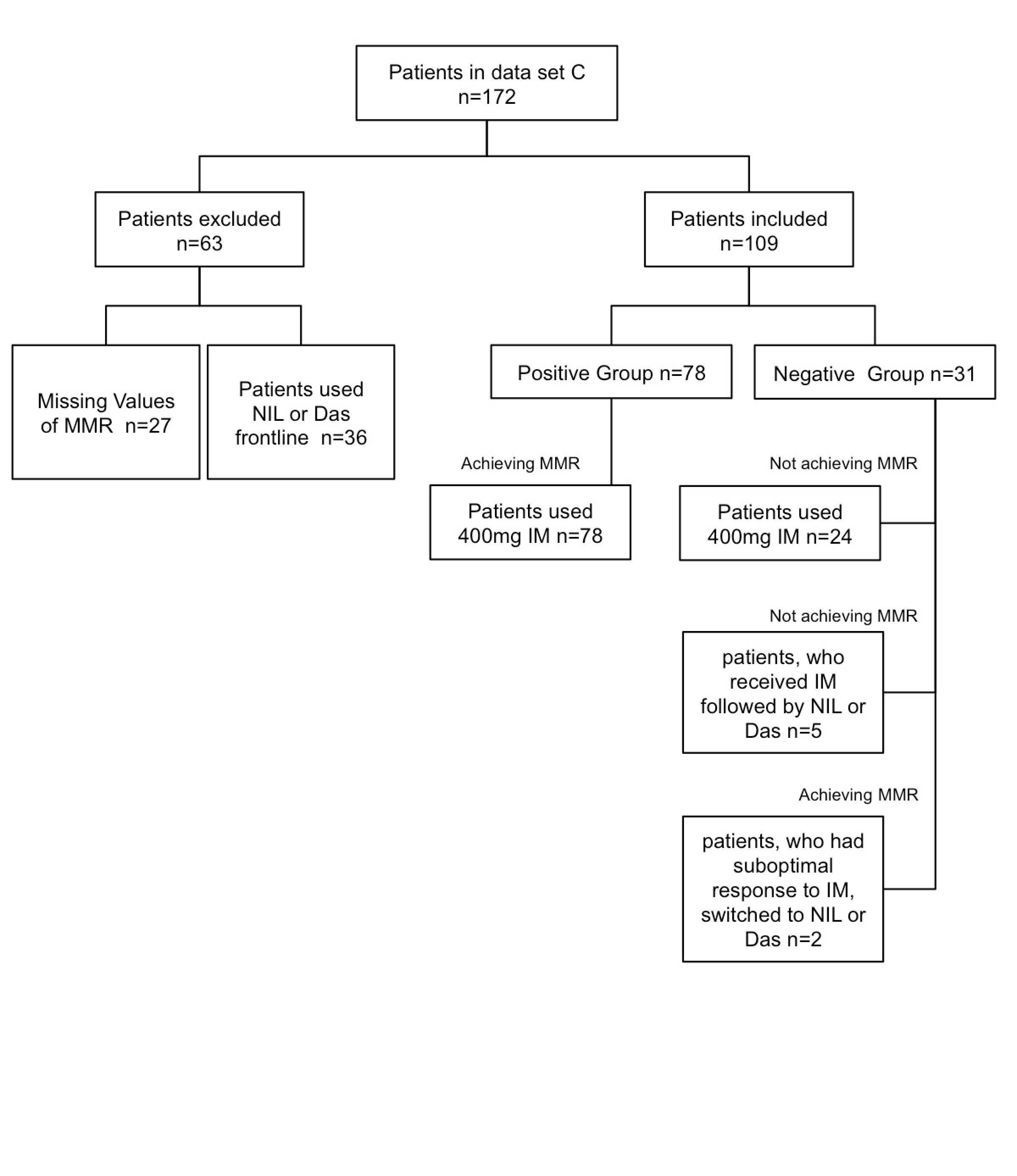

Supplement: S1 File — (ZIP) [file pone.0168947.s002.zip › S1_File/S1_FigD.tif]
